# Small Open Reading Frame-Encoded Micro-Peptides: An Emerging Protein World

**DOI:** 10.3390/ijms241310562

**Published:** 2023-06-23

**Authors:** Xiaoping Dong, Kun Zhang, Chengfeng Xun, Tianqi Chu, Songping Liang, Yong Zeng, Zhonghua Liu

**Affiliations:** 1 National & Local Joint Engineering Laboratory of Animal Peptide Drug Development, College of Life Sciences, Hunan Normal University, Changsha 410081, China; 2 Peptide and Small Molecule Drug R&D Platform, Furong Laboratory, Hunan Normal University, Changsha 410081, China; 3 The State Key Laboratory of Developmental Biology of Freshwater Fish, College of Life Science, Hunan Normal University, Changsha 410081, China

**Keywords:** small (short) open reading frame, sORF, sORF-encoded peptides, SEPs, micro-proteins, peptidome, coding potential prediction

## Abstract

Small open reading frames (sORFs) are often overlooked features in genomes. In the past, they were labeled as noncoding or “transcriptional noise”. However, accumulating evidence from recent years suggests that sORFs may be transcribed and translated to produce sORF-encoded polypeptides (SEPs) with less than 100 amino acids. The vigorous development of computational algorithms, ribosome profiling, and peptidome has facilitated the prediction and identification of many new SEPs. These SEPs were revealed to be involved in a wide range of basic biological processes, such as gene expression regulation, embryonic development, cellular metabolism, inflammation, and even carcinogenesis. To effectively understand the potential biological functions of SEPs, we discuss the history and development of the newly emerging research on sORFs and SEPs. In particular, we review a range of recently discovered bioinformatics tools for identifying, predicting, and validating SEPs as well as a variety of biochemical experiments for characterizing SEP functions. Lastly, this review underlines the challenges and future directions in identifying and validating sORFs and their encoded micropeptides, providing a significant reference for upcoming research on sORF-encoded peptides.

## 1. Introduction

According to the ENCODE database, about 2–3% of the human genome is composed of protein-coding genes, and more than 80% are viewed as ncRNAs [1,2]. With the advanced development of high-throughput sequencing technology, more and more diverse ncRNAs have been discovered to be involved in numerous essential biological processes, such as genomic regulation [3], environmental responses [4], and body development [5]. Generally, ncRNAs are classified into long non-coding RNAs (lncRNAs), small RNAs (miRNAs), piwi-interacting RNAs (piRNAs), circular RNAs (cirRNAs) and others. They were initially considered “transcriptional noise” [6,7]. However, research has reversed the view that ncRNAs represent “junk” transcription products [8]. One or more short open reading frames (sORFs), which rarely use AUG as the start codon, may be present in these ncRNAs. The majority are initiated by near-homologous codons (meaning codons that differ from AUGs by one nucleotide), such as CUG, GUG, UUG, and ACG [9]. These sORFs may encode small proteins with less than 100 amino acids, and various professional terms have been used to describe these proteins, such as micropeptides, small peptides, microproteins, sORF-encoded peptides (SEPs), etc.

Typically, an ORF is defined as a segment of conserved and non-overlapping nucleotide triplets (codons) that can be translated into a functionally annotated protein [10]. Eukaryotic messenger RNAs (mRNAs) usually contain a main ORF that produces protein-coding regions. However, the traditional genetic rules, such as amino acid conservation and homology, the absolute requirement for a starting codon (methionine), and the minimum translation length, have greatly limited the identification of transcripts with non-canonical protein-coding capabilities. Therefore, we regarded these proteins encoded by previously neglected open reading frames with fewer than 300 nucleotides (nt) as sORF-encoded peptides (SEPs). SEPs are biologically active molecules that range from highly conserved to primate-specific [11], implying that they perform both basal and species-specific functions. To date, SEPs have been found to function in a variety of biological processes, including embryogenesis [12,13,14], myogenesis [15,16,17], cellular metabolism [18,19], inflammation [20,21,22], and carcinogenesis [23,24,25,26].

Due to the limitations of the conservation screening mechanism and detection sensitivity, SEPs with a small molecular weight and a low expression abundance are often overlooked, which may lead to many crucial regulatory mechanisms being “hidden”. Therefore, it is challenging to determine the potential functional roles of such micro-proteins. With the increasing interest in SEPs, a large number of new ORF translation products have been identified and validated. In summary, this reflects the diversity of SEPs under different physiological conditions. It is urgent to identify and characterize their functional roles, which may reveal many new molecules involved in regulatory mechanisms.

## 2. Localities and Characteristics of sORFs and SEPs

**Figure 1:**
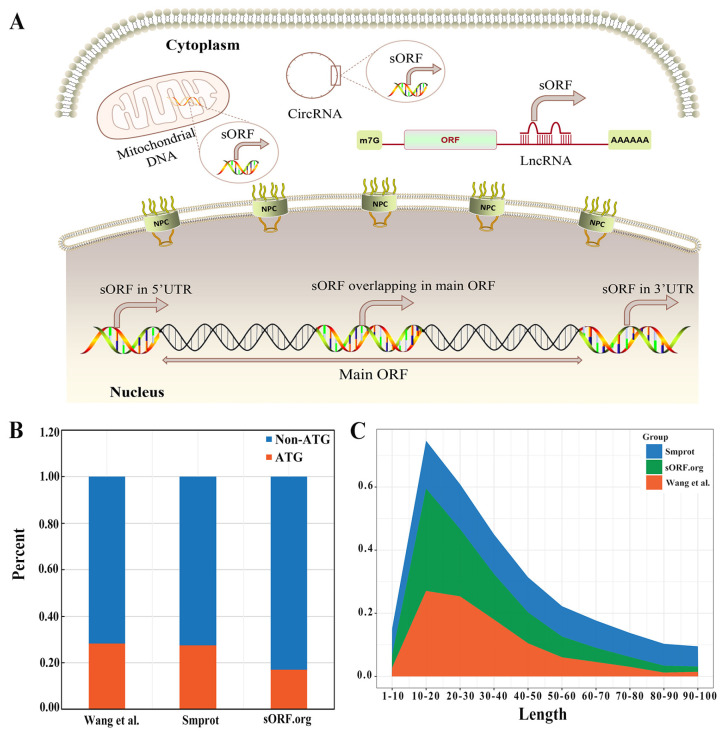
Localities and characteristics of sORFs and SEPs. (**A**) Examples of sORFs within coding transcripts (5′ UTR, CDS, 3′UTR) or even within non−coding RNAs (circRNA, lncRNA, mitochondrial RNA); (**B**) the percentage of ATG and non-ATG start codons in public databases and a representative study; (**C**) the AA length distribution of SEPs.

In recent years, extensive translations of sORFs at genomic locations in animal, plant, fungal, and bacterial species have been revealed based on high-throughput next-generation sequencing technologies [27,28,29]. Theses sORFs can be located within coding transcripts such as 5′ UTR (5′ untranslated regions), CDS (coding sequences), 3′ UTR (3′ untranslated regions) or even within non-coding RNAs, such as long non-coding RNAs (lncRNAs), cirRNAs, and mitochondrial RNAs (mtRNA) (Figure 1A). sORFs are essentially hidden genomic features in the organism [30]. Therefore, it is possible to find new proteins with interesting functions.

As we all know, non-coding RNAs (ncRNA) include long non-coding RNAs (lncRNAs, longer than 200 nt) and small non-coding RNAs (sncRNAs). Many important physiological processes have been found to be regulated by the micro-peptides translated by lncRNAs [21,31,32]. Traditionally, upstream open reading frames (uORFs) are located upstream of protein-coding genes and are considered as cis-acting elements for downstream expression through a mechanism similar to competitive translation [33]. Beyond these, recent studies have shown that uORFs can encode functional micro-peptides. Like uORFs, small peptides encoded by dORFs (downstream open reading frames) are usually not conserved, and the effects of the dORFs are not dependent on the small peptides, but on the translational activity of the dORFs themselves [34]. CircRNAs often function as miRNA sponges and play roles in transcriptional regulation and protein binding. CircRNAs have been shown to have the ability to translate in recent years [35,36,37]. In addition, sORF-encoded peptides (SEPs) were discovered in pseudogenes [38] as well as in intergenic regions [39].

Bioinformatic predictions and MS-based proteomics approaches have been used to predict and identify SEPs with different lengths and start codons. Wang et al. identified 1682 peptides from 2544 human sORFs in Hep3B cells using a de novo approach combined with RNA-Seq [40]. Several online sORF databases such as Smprot [41], sORF [42], and OpenProt [43] have been constructed. Unexpectedly, a large proportion of SEPs are translated with non-AUG initiation codons. Usually, alternative start codons only differ from AUG by one nucleotide (e.g., CUG, GUG and UUG). It has been shown that these non-classical start codons are homologous to the classical start codon ATG, which is often located near the Kozak sequence [44]. Another theory suggests that the non-classical start codon of sORFs is derived from the RNA editing of post-transcriptional mRNA, which converts uracil (U) to cytosine (C) in the transcription product initiation codon AUG by the action of RNA editing enzymes, thus converting the classical start codon to a homologous non-classical codon and regulating the translation efficiency of sORFs [45]. Additionally, it was discovered that SEPs have similar length ranges, but slightly different distributions. A possible explanation for this variation is the use of different scoring algorithms and computational software (Figure 1B,C). However, these SEPs with less than 100 amino acids in length deserve further investigation.

## 3. Ribosome Profiling (Ribo-Seq) for Identification of SEPs

**Figure 2:**
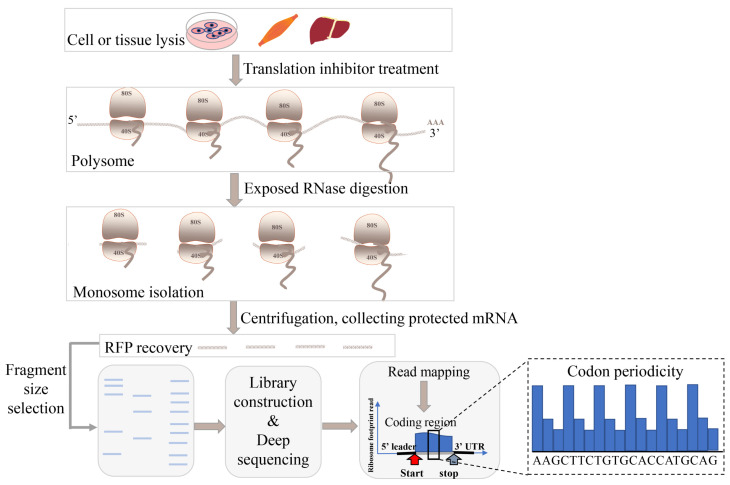
Ribosome profiling process, where ribosome footprints are obtained for deep sequencing. Isolation of ribosome-bound mRNAs is conducted through treatment of non-specific nucleases such as RNase I. Ribosome footprints (showing positioning between start and stop codon of gene) are then used for library generation and deep sequencing.

Ribosome profiling is an emerging technique that uses deep sequencing to monitor in vivo translation and provides a systematic method for the experimental annotation of coding regions. The whole workflow is designed to degrade the RNA that is not protected by ribosomes using RNA enzymes before centrifuging to separate the ribosome-protected mRNA fragments. These 30 nt footprints can be directly mapped to the original mRNA by deep sequencing and further used to pinpoint the precise location of the ribosomes during translation (Figure 2). However, Wilson et al. demonstrated that not all sORFs bound to ribosomes are translated [46]. In order to separate the mRNA bound to multiple ribosomes and distinguish single ribosome–mRNA complexes that are not translated, poly-Ribo-Seq was developed. The technology provides more concrete evidence of active translation.

Ribosome profiling has proven to be a powerful technique to explore the translation potential of sORFs by using multiple pipelines (Table 1). Previous studies have presented an experimental and analytical framework for the systematic identification and quantification of translation based on ORF-RATER [47]. RiboTaper can detect regions of active translation based on three-nucleotide periodicity [48]. Calviello et al. used RiboTaper to identify 218 novel proteins in Chinese hamster tissue and CHO cell lines [49]. As the technology has advanced and matured, more analytical tools such as Ribowave [50], RibORF [51], and RiboCode [52] have been used to support the reference database construction for mining SEPs from MS data.

While ribosome sequencing can provide a landscape of ribosome occupancy throughout the transcriptome, its sequencing data can provide information on where translation occurs and quantitative information, such as how much of the region is occupied by ribosomes. Ribo-Seq does have some limitations. Firstly, Ribo-Seq requires the rapid suppression of translation to capture ribosome snapshots in a specific physiological state, leading to possible inaccuracies in data collection [53]. Secondly, the technique requires inferring the speed of protein synthesis, but it is accurate based on the assumption that all of the ribosomes have completed translation. In fact, translation pauses or discontinuities may also occur under certain conditions, such as starvation [54]. Thirdly, contaminated RNA fragments (including non-coding RNAs or ribosome–protein complexes) may migrate during gradient centrifugation, be found in cDNA libraries, and lead to misreading in translation. Lastly, the generated RPFs with 30 bp are not easy to map [55]. Because these RPFs are often too short to provide unique mapping information, when these short sequences are aligned to the reference genome or transcriptome, they may align to multiple locations due to the presence of repetitive or highly similar sequences. This makes it difficult to determine the precise location of the mRNA on the ribosome during translation. Moreover, the short length of these sequences can also lead to sequencing errors, which further complicates the mapping process. In addition, these short sequences may not provide enough context to accurately identify the frame of translation, which can affect downstream analysis and interpretation.

**Table 1:** Evaluation tools/software of sORF translation by ribosomal profiling methods.

Tool	Feature	Availability	Ref.
ORF-RAETER	Translated ORF identification and quantification based on linear regression.	https://github.com/alexfields/ORF-RATER.githttps://github.com/alexfields/ORF-RATER	[47]
Ribo-TISH	A comprehensive toolkit for analyzing TIs and predicting putative ORFs.	https://github.com/zhpn1024/ribotish.git	[56]
Ribowave	ORF prediction, protein abundance estimation, TE calculation, and ribosomal frameshift identification based on wavelet transform.	https://ribowave.ncrnalab.org/	[50]
RiboORF	ORF identification based on the arrangement of the ribosome A site, the 3 nt periodicity, and the consistency between codons.	https://github.com/zhejilab/RibORF.git	[51]
RiboHMM	ORF identification based on the total abundance and periodic codon structure in RPF.	https://github.com/djf604/RiboHMM.git	[57]
ORFquant	ORF prediction and quantification based on multi-taper method.	https://github.com/lcalviell/ORFquant/releases/tag/1.02	[49]
Ribotricer	ORF prediction based on three-nucleotide periodicity.	https://github.com/smithlabcode/ribotricer/releases/tag/v1.3.3	[58]
PRICE	Resolving overlapping sORFs and noncanonical translation initiation based on machine-learning model.	https://github.com/erhard-lab/price.git	[59]
Ribocode	De novo assembly and annotation for translatomes based on Wilcoxon signed-rank test.	https://github.com/xryanglab/RiboCode.git	[52]
RiboTaper	ORF identification based on the characteristic three-nucleotide periodicity of Ribo-Seq.	https://ohlerlab.mdc-berlin.de/software/RiboTaper_126/	[48]
RP-BP	ORF prediction based on unsupervised Bayesian approach.	https://github.com/dieterich-lab/rp-bp.git	[60]
SPECtre	ORF prediction based on 3 nt periodicity.	https://github.com/mills-lab/spectre.git	[61]
RiboToolkit	Ribo-Seq web application for analysis and implementation of a full ORF prediction pipeline.	http://rnainformatics.org.cn/RiboToolkit/analysis.php	[62]
GWIPS-Viz	Online web server for visualizing processed Ribo-Seq data.	https://gwips.ucc.ie/	[63]
Trips-Viz		https://trips.ucc.ie/	[64]

## 4. Peptidomic-Based Methodology for Identification of SEPs

The MS-based technique is the most direct evidence that sORFs can be translated. As with traditional bottom-up proteomics studies, the identification workflow for SEPs includes sample extraction and enrichment, digestion and separation, MS data collection, and analysis.

### 4.1. Sample Extraction and Enrichment

**Figure 3:**
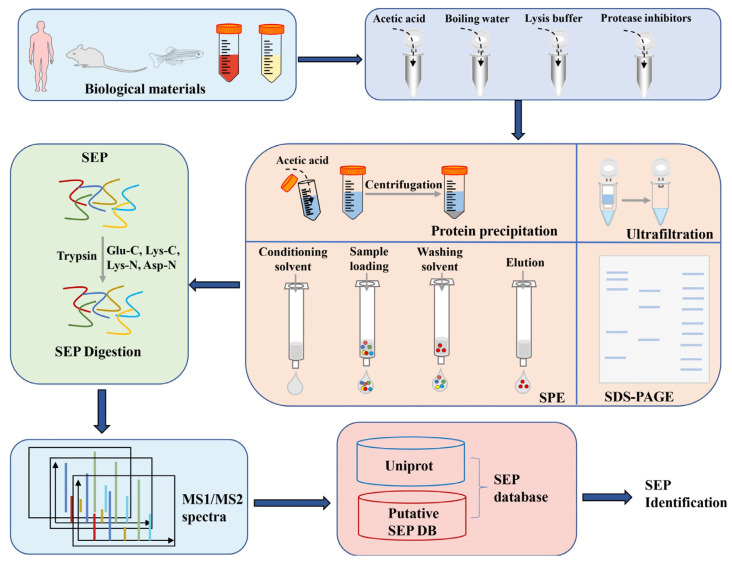
General workflow of a peptidomics approach for identifying SEPs. SEPs are extracted from complex biological samples, enriched by different methods such as protein precipitation, ultrafiltration, and SPE, then digested with trypsin (or multiple enzymes). The tryptic peptides are subjected to fractionation, MS data acquisition, and data analysis to identify SEPs.

The first critical step for SEP identification is extracting SEPs from complex biological matrices while ensuring their integrity (Figure 3). SEPs with a small size and a low molecular weight are difficult for peptidases to hydrolyze, and they may not have any sites for protease digestion or may be covered by undesired protein degradation products [65]. SEP extraction is, therefore, more challenging than that of proteins. Previous studies have tried various methods to ensure the integrity of SEPs, such as heating samples in boiling water or lysis buffers, using an ultrasonic treatment, or adding protease inhibitors to inhibit peptidase and protease activity [66,67]. However, some polypeptides, such as peptidases or protease inhibitors, can interfere with the subsequent analysis of SEPs. Therefore, some studies have proposed alternative methods, such as inducing protein precipitation with hydrochloric acid or acetic acid, which not only effectively prevent the degradation of SEPs, but also do not interact with polypeptide enzymes [67]. Therefore, the treatment of biological samples is a key step in extracting SEPs. The stability of biological samples and the objectives of the research should guide the selection of the appropriate extraction techniques.

The enrichment of SEPs is mainly used to separate the target peptides from other proteins in the same sample, thus reducing the complexity of the sample. These separation methods frequently depend on various physical properties of the sample, such as the size, hydrophobicity and charge. Organic solvent (acetonitrile [40], acetone, methanol [68], trichloroacetic acid, and acetic acid [67]) precipitation could effectively retain low-molecular weight proteins in the supernatant liquid. In another endeavor, sequential precipitation and dehydration (SPD) based on a methyl tert-butyl ether/methanol/water system was used to successfully detect 129 proteins smaller than 30 kDa from human plasma, showing a good sensitivity and reproducibility. Size exclusion approaches have also been extensively used for protein isolation. High-molecular weight proteins can be kept on filter by using 10 or 30 kDa molecular weight cut-off (MWCO) ultrafiltration membranes [69,70]. However, this membrane-based technique has several drawbacks, including the potential blockage of membrane pores due to concentrated macromolecules, the non-specific binding of small proteins to hydrophobic surfaces, and time-consuming processes. C8-SPE is another method based on hydrophilic and hydrophobic properties of SEPs [66]. It was reported that a combination of these methods may be able to identify more SEPs [71].

### 4.2. Digestion of Samples for Mass Spectrometry

Sample digestion is a crucial component as well. SEPs tend to be short peptides with less than 100 amino acids and fewer arginine and lysine residues than other peptides. The single trypsin may cause cleavage failure or produce fewer trypsin peptides, reducing the sequence coverage and making it impossible to be detected by MS [72]. Due to the sequential digestion and complementary cleavage specificity, multi-protease digestion combined with trypsin and other proteolytic enzymes such as Glu-C (endoproteinase Glu-C), Lys-C (endoproteinase Lys-C), Lys-N (endoproteinase Lys-N), Asp-N (endoproteinase Asp-N), Arg-C (endoproteinase Arg-C), and chymotrypsin has been shown to enhance micro-peptide recognition effectively [73,74].

To date, mass spectrometry is still the only method available for the direct detection and quantification of SEPs. Data dependent acquisition (DDA) is the most widely used for MS acquisition analyses. In the past five years, thousands of SEPs have been identified using DDA from different species, including humans [75], *E. coli* [76], and plants [77]. The method is suitable for peptides ranging from 8 to 25 amino acids, but SEPs cannot produce fragments in this range due to the absence of required cleavage sites [78]. On the other hand, due to the small size of micro-peptides, only one peptide can be used for a peptide spectrum match (PSM) [79,80], which may increase the false detection rate in SEP identification [81,82]. Fortunately, it was discovered that targeted proteomics is a promising method with higher confidence. The expression of SEPs was tracked using parallel reaction monitoring (PRM) and data independent acquisition (DIA) in different biological samples [83]. In addition to simultaneously breaking up all precursor ions, DIA also preserves data that can be analyzed repeatedly in silico using various spectral libraries. Pak et al. [84] reported that the number of immune peptides identified had increased by almost three-fold using DIA. By selectively detecting particular peptides, parallel reaction monitoring (PRM) aims to achieve the relative or absolute quantification of a target protein or peptide [85]. These approaches are expected to benefit substantially from further improvements in analytical pipelines.

### 4.3. Database Construction for SEPs

With the accumulation of encoded sORFs and their corresponding SEPs, numerous publicly accessible repositories devoted to sORFs have been developed for SEP identification (Table 2).

Both SmProt [86] and sORF.org [42] are well known to researchers. SmProt collects small proteins from eight species, including *Homo sapiens*, *Mus musculus*, *Rattus norvegicus*, *Drosophila melanogaster*, *Danio rerio*, *Saccharomyces cerevisiae*, *Caenorhabditis elegans*, and *Escherichia coli*, that have been identified through ribosomal analysis data, the literature, and mass spectrometry (MS) [86]. SmProt also includes information about the sequences, genomic locations, coding potential assessment, function, and other characteristics of the collected small proteins [41]. sORFs.org and OpenProt assess the identity of protein sequences based on BLASTp scores. Specific details about the target micro-peptide, such as the species, chromosome number, starting codon, and sORF attributes, can be requested through sORFs.org [42]. While OpenProt proposes a comprehensive annotation of predicted coding sequences on all transcripts, it provides obvious evidence for the expression of novel protein products [43]. The combination of these public databases could speed up the identification of micro-proteins. MetamORF only contains sORF data for *Homo sapiens* and *Mus musculus* [87]. ARA-PEPs [88] and PsORF [88] are comprehensive web servers dedicated to searching, browsing, visualizing, and downloading plant sORF-encoded peptides. These resources make it simple to construct reference databases for identifying and analyzing SEPs.

The combination of private databases and public databases is also a good choice. Generally, custom databases rely on a six-frame translation of the genome sequence to produce a reference database with all potential SEPs. Many researchers have combined custom databases and public databases such as Ensembl, RefSeq, and UniProtKB [44,89] for mining new SEPs [73,90,91,92]. However, it is undeniable that these databases contain a large number of pseudo-sequences, which reduces the confidence of peptide profile matching (PSM) and makes it challenging to detect SEPs with a low abundance [83].

The de novo sequencing of MS data is a library-independent method that deciphers protein or peptide sequences only from the spectrum without any genomic reference information [93]. Chen et al. and Wang et al. identified hundreds of SEPs using PEAK [94] and pNovo3 [40], respectively. However, it should be noted that many de novo peptides cannot be matched to any ORFs using the algorithms available today. This may be due to rare starting codons, mutations, or splicing, or it might require improved gene mapping algorithms to dock de novo sequencing.

### 4.4. Bioinformatic Tools for sORF and SEP Predictions

With the advance of high-throughput sequencing technology, many functional SEPs have been found. It is necessary to re-evaluate the coding potential of sORFs. However, the identification and prediction of sORFs with coding capabilities have become more complex due to the relative lack of consensus features. A wide variety of computational tools have been developed for predicting and distinguishing non-coding and coding transcripts based on nucleotide composition, codon substitution, machine-learning algorithms, and others (Table 3).

### 4.5. Prediction of Coding Potential and Sequence Conversion of sORFs

As an original tool, the coding region identification tool invoking comparative analysis (CRITICA) compares genomic regions across multiple species to identify conserved non-coding regions that are likely to contain functional sORFs [95]. Another computational tool, the coding potential calculator (CPC), calculates a coding potential score based on features such as ORF length, ORF coverage, and conservation [96]. Therefore, it may miss some functional sORFs that are not conserved across species. Y. et al. used CPC to predict the coding potential of the lncRNA DLEU1 and found that DLEU1 encodes a membrane-channel small peptide that affects glioma cell development, invasion, and metastasis [115].Other tools such as RNAcode [102], micPDP [98], and phyloCSF [99] make use of a different principle known as codon substitution. The criteria used by PhyloCSF to identify sORFs include the presence of an ORF with a length of at least 30 nucleotides and evidence of purifying selection across multiple species. Mackowiak et al. predicted 354 conserved sORFs in the lncRNAs based on Ribo-Seq and PhyloCSF, and validated 22 peptides using MS spectral data [116]. ORF finder is a tool that identifies ORFs in nucleotide sequences. It does not specifically predict sORF coding potential, but rather identifies all ORFs, including potentially functional and non-functional ones. Growing evidence points to machine-learning (ML) algorithms as another options for sORF coding potential prediction, such as DeepCPP [112] and MipepID [114]. In particular, MipepID is designed specifically to predict the coding potential of sORFs. Fesenko, Igor et al. identified thousands of evolutionarily conserved smORFs in Physcomitrium patens using MipepID [117]. Therefore, CRITICA is the most effective tool for identifying functional sORFs in some species, while PhyloCSF and CPC/CPC2 may be better suited for identifying conserved and novel sORFs, respectively. ORF finder is a useful tool for identifying all ORFs in a sequence, but may identify many false positive sORFs. The emergence and development of these tools reflects the endeavor to study sORFs.

### 4.6. Prediction Tools Related to SEP Structure

In addition to predicting the coding potential of sORFs, it is necessary to perform structurally related predictions of their functional micro-peptides. Currently, several tools, such as TMHMM [118], SignalP [119], ProtScale [120], and AlphaFold2 [121], also have been used to predict the localization, transmembrane regions and protein structure of the target micro-peptides. TMHMM is currently the most effective and best-performing method for the prediction of transmembrane segments of micropeptides [118]. SignalP 5.0 predicts the presence of signal peptides and the location of their cleavage sites, which helps researchers to understand the mode of action of micro-peptides. A tool called ProtScale makes it possible to compute and represent the profile produced by any amino acid scale, and it serves as a guide for the identification of micro-peptide transmembrane regions. Additionally, SWISS-MODEL [122] and AlphaFold2 can be applied to generate reliable 3D protein models, which can enable an in-depth exploration of the biological functions and structural features of micro-peptides. Zhou et al. used several functional tools, including IAMPE [123], Phobius [124], Pfam [125], TMHMM, and ProtScale, to analyze these candidate micro-peptides, indicating that an SEP (SEP068184) may regulate oxidative resistance through involving metabolic pathways and interacting with cytoplasmic proteins in *Deinococcus radiodurans* [126]. Moreover, there are additional resources available for researchers to investigate the specific physical and chemical properties or functions of SEPs, including ProtParam, BUSCA [127], and SOPMA [128].

## 5. Experimental Validation of Micro-Peptide Coding Potential and Function

Recent studies have identified thousands of additional components of the proteome. The majority of these components are micro-peptides that sORFs in noncoding regions translate. Although Ribo-Seq, bioinformatics prediction and peptidomics are mostly sufficient for the requirements of high-throughput micro-peptide screening and discovery, corresponding biochemical experiments are necessary to prove their true existence.

### 5.1. Validation of Translation of sORFs from Putative SEPs

Firstly, antibodies can specifically recognize a target protein and are a direct and highly sensitive method for detecting the endogenous expression of SEPs in tissues or cells. Li et al. detected the endogenous expression of MIAC by preparing monoclonal antibodies to MIAC [129]. However, these SEPs have a low antigenicity and contain transmembrane structural domains, which largely limit the selection of immune epitopes and make it still extremely challenging to produce specific and effective antibodies against peptides [19,30,130,131].

For SEPs without corresponding antibodies, an epitope tag is another option for detecting the endogenous expression of SEPs. In order to create a fusion protein that contains both SEPs and protein tags, fluorescence (GFP) or epitope tags can be inserted into the candidate SEP sequence using CRISPR/Cas9-mediated gene-editing techniques. The presence of the SEP is then confirmed by Western blotting and the immunoprecipitation of these fusion proteins [17,132,133,134,135]. To determine whether the sORF in the CASIMO1 transcript was translated into a micro-peptide, Schwarz et al. inserted a FLAG tag at the C-terminus of the CASIMO1 coding sequence and detected the expression of CASIMO1-FLAG by an anti-FLAG antibody [136]. Nevertheless, if these SEPs are relatively small, additional peptide fractions may alter their physiological properties, localization, or protein interactions [137]. There are a variety of different epitope tags, including FLAG [136], APEX [137], HA [138], V5, fluorescent proteins, etc. It is essential to choose the appropriate epitope tag according to the characteristics of the SEP.

In addition to micro-peptide validations based on antibody studies, the sORF coding potential may also be determined using in vitro translation assays [15,138,139]. The experiment requires additional experiments to verify the release of the SEP [130], such as the introduction of frameshift mutations, which are used as negative controls to further verify the results.

### 5.2. Demonstration and Validation of Biological Relevance for SEPs

The above experiments validated the capacity of sORFs for translation; however, molecular experiments are needed to determine the potential function of the identified SEPs. Most of these methods are similar to determining the common protein function, but are relatively complex. The CRISPR/Cas9 technique is frequently employed to detect the effects of SEPs on phenotypes [11,15,140]. Special vectors for SEPs, such as loss-of-function (e.g., knockdown or knockout) or gain-of-function (e.g., overexpression or activation) vectors, can be designed for cell transfection to observe the effect on the phenotype, further inferring the function of SEPs. Fu et al. identified a highly conserved transmembrane micro-peptide called NEMEP by CRISPR/Cas9, providing a clear example of the direct functional effect of altered glucose metabolism on cell fate decisions [138]. However, not all SEPs can benefit equally from the functional validation experiments of CRISPR/Cas9. When translatable sORFs exist in lncRNAs, the validation experiments often need to be achieved using frameshift or start codon mutations, which not only selectively inhibit micro-peptide expression, but also have no impact on lncRNAs [30,141].

Synthesizing the corresponding peptides is another way to confirm the function of SEPs. Pauli et al. successfully applied this method to demonstrate that the synthesized toddler peptide has the same phenotype as mRNA overexpression [142]. In addition, rescue experiments can be performed to verify whether the sORFs or SEPs are responsible for regulatory functions [143]. After the functions of SEPs are certified, the underlying regulatory mechanism behind these SEPs becomes an urgent issue for subsequent research. MS and immunoprecipitation can be used to identify specific protein complexes. The function or pathway of the co-purification protein can then be used to deduce the function of the micro-peptides [132].

The functional verification of the SEPs encoded by UTR regions is relatively difficult. To characterize the biological relevance of uORF-encoded micro-peptides, uORF perturbations may affect the stability of the main ORF, further confusing the process for revealing the uORF function. In a previous study, antisense oligonucleotides (ASOs) against uORF were used to up-regulate the CDS expression, which was a more novel strategy [144]. Although the underlying regulatory mechanism is unclear, uORF-targeted ASO has been used to restore downstream gene expression by regulating the efficiency of ribosome initiation [145]. Thus, ASO is suitable as a functional tool to assess the effect of a given uORF on the CDS expression.

## 6. Biological Functions of sORF-Encoded Peptides: Relevant Examples

To date, many SEPs have been identified and characterized, and they are involved in a variety of physiological processes, such as calcium homeostasis, metabolism, muscle development, substance degradation, gene transcription and translation regulation, and cancer development.

**Figure 4:**
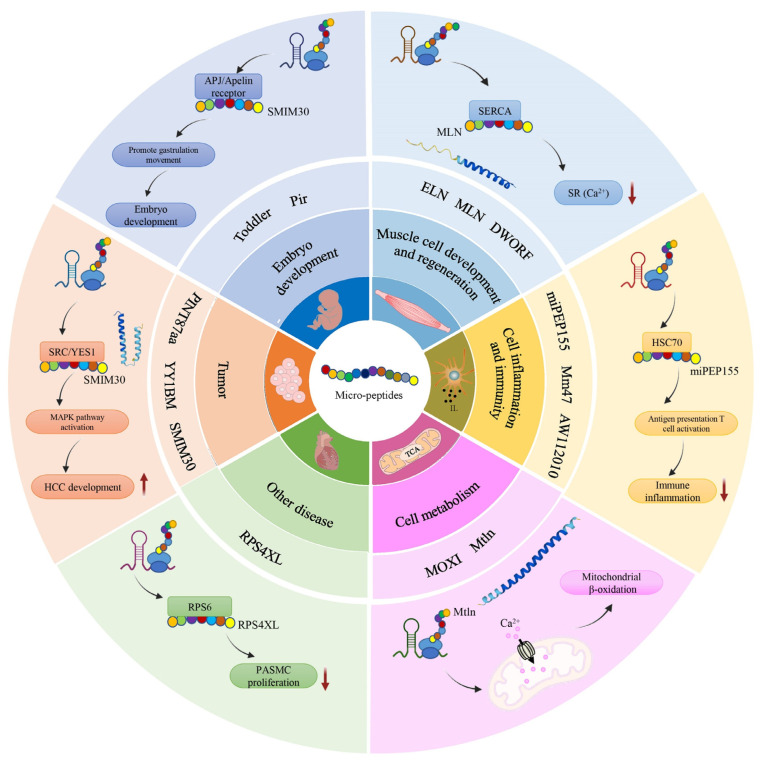
Various biological roles of micro-peptides encoded by putative ncRNAs. From the outer layer to the inner layer are the specific molecular mechanisms of representative SEPs, representative SEPs and their corresponding biological functions, respectively. Upward arrows indicate promotion, downward arrows indicate inhibition.

For example, the lncRNA MIR155HG was the subject of extensive research for its contribution of miRNA products (miR-155) in inflammation and adaptive immune responses. It was reported that the human lncRNA MIR155HG encoded the 17-amino acid micro-peptide miPEP155 (P155). MIR155HG is highly expressed by inflamed antigen-presenting cells, leading to the discovery that P155 interacts with the adenosine 5′-triphosphate binding domain of heat shock cognate protein 70 (HSC70), a chaperone required for antigen trafficking and presentation in dendritic cells (DCs). P155 modulates major histocompatibility complex class II-mediated antigen presentation and T cell priming by disrupting the HSC70-HSP90 machinery [21]. Here, a summary of more SEPs and their biological functions is provided (Table 4 and Figure 4).

## 7. Conclusions and Future Perspectives

Tradition dictates that genes encode only one protein and that transcripts without a main ORF are non-coding. In this review, we revealed a new research area: ncRNAs that can encode peptides or small proteins. We elaborated on the location of sORFs in the genome, the identification of encoded peptides, and the analytical procedures and subsequent methods for the validation of biological function mechanisms, revealing previously unrecognized complexity in the proteome. In recent years, SEPs have been found to exist and play important biological regulatory roles in most species, including humans, mice, rats, zebrafish, flies, yeast, and *Escherichia coli*. In addition, with a relatively small size, a tissue-specific expression pattern, and a low cytotoxicity, SEPs will be a new resource pool for screening anti-tumor peptides or protein drugs, and they will play an important role in accurate diagnoses, precise classifications, precise treatments and tumor prognoses. So far, SEPs have been found to have significant antitumor functions by inhibiting cancer metabolic reprogramming, oncogenic protein stability, and oncogenic-related pathways, making them new therapeutic targets for clinical applications. However, SEPs are characterized by short peptide fragments, a small molecular weight, and a low expression abundance, which may cause difficulties in the extraction and synthesis of micro-peptide drugs and inaccurate identification of relevant detection technologies. Therefore, continued advancements in the field will depend on clever experimental designs and further optimization of the relevant technology.

Although many SEPs with coding potential have been characterized in the last few years, the following crucial and urgent questions still need to be answered: (1) How can a sufficient number of SEP samples be obtained for a more thorough investigation? The small molecular weight and low expression abundance of SEPs make it difficult to obtain active samples via genetic engineering; (2) The annotation of SEPs is primarily based on phylogenetically conserved analyses, but how else can new peptides be validated in the absence of sequence conservation? How do the different SEPs work? (3) Given the growing evidence that not all peptides initiate translation by AUG, how do we begin to validate the true translation initiation codons with the current genome annotations of uORFs and main ORFs? Do initiation codons other than AUG codons employ a different mechanism? (4) Only the human and a few animal models are included in the current database of species annotated for SEPs. The inter-species differences have led to many databases being insufficient to meet the requirements of micro-peptide research at this stage, so the establishment of functional annotation databases is particularly important. There is no doubt that the mechanism of sORF-encoded micro-peptides will spark a new research boom and advance the life sciences; they will provide new insights for future investigations to unravel intricate physiological processes and diagnose diseases in living organisms.

## Figures and Tables

**Table 2:** Commonly used databases for micro-peptide research.

Database	Resource	URL	Function
SmProt	Human and other species	http://bigdata.ibp.ac.cn/SmProt/index.html	A reliable repository with a comprehensive annotation of small proteins derived from ribosome profiling, literature, mass spectrometry (MS), etc.
sORF.org		http://www.sorfs.org (The website link is temporarily unavailable)	A public repository for sORFs identified both from experiments and in silico (based on various bioinformatics tools) to allow researchers to examine individual sORFs or to perform searches.
Openprot		https://www.openprot.org/	Contains all known proteins (RefProts), novel predicted isoforms (Isoforms) and novel predicted proteins from alternative ORFs (AltProts), supporting the annotation of thousands of predicted ORFs.
nORF.org		https://norfs.org/home	Combines existing databases such as sORFs.org, OpenProt, and openCB to provide more comprehensive information.
MetamORF		https://metamorf.hb.univ-amu.fr/	Provides unique sORFs identified in the human and mouse genomes with both experimental and computational approaches.
FuncPEP		https://bioinformatics.mdanderson.org/Supplements/FuncPEP	A new database of functional ncRNA encoded peptides, containing all experimentally validated and functionally characterized ncPEPs.
PsORF	Plant	http://psorf.whu.edu.cn/	A web collection resource of small open reading frames (sORFs) for 35 plant species to provide translation evidence and information on the evolutionary conservation of those small ORFs.
ARA-PEPs		https://github.com/rashmihazarika/ARA-PEPs.git	Specific to all putative sORF-encoded peptides in Arabidopsis thaliana.

**Table 3:** Some bioinformatic resources for coding potential of sORF.

Tools	Data Requirement	Principle Utilized	URL	Ref.
CRITICA	Whole genome	Nucleotide sequence composition	http://rdpwww.life.uiuc.edu (The website link is temporarily unavailable)	[95]
CPC/CPC2	RNA-seq	Nucleotide composition, sequence similarity	http://cpc2.gao-lab.org/	[96]
PLEK	RNA-seq	Kmer composition of sequence	https://sourceforge.net/projects/plek	[97]
micPDP	RNA-seq	Codon conservation	https://github.com/Drmirdeep/micpdp.git	[98]
PhlyoCSF	RNA-seq	Codon substitution, nucleotide sequence similarity	http://compbio.mit.edu/PhyloCSF	[99]
PhastCons	Whole genome	Nucleotide composition	http://compgen.cshl.edu/phast/	[100]
sORF finder	Any nucleotide sequence	Nucleotide composition	http://evolver.psc.riken.jp/ (The website link is temporarily unavailable)	[101]
RNAcode	RNA-seq	Codon conservation	https://github.com/ViennaRNA/RNAcode.git	[102]
CNCI	RNA-seq	Nucleotide composition	https://github.com/www-bioinfo-org/CNCI.git	[103]
CPAT	RNA-seq	Permutation-free logistic regression model	https://wlcb.oit.uci.edu/cpat	[104]
CNIT	RNA-seq	Nucleotide composition	https://github.com/www-bioinfo-org/CNCI.git	[105]
ORF finder	Whole genome	Nucleotide composition	https://www.ncbi.nlm.nih.gov/orffinder/	[106]
iSeeRNA	RNA-seq	Machine-learning model	http://www.myogenesisdb.org/iSeeRNA (The website link is temporarily unavailable)	[107]
COME	RNA-seq	Machine-learning model	https://github.com/lulab/COME.git	[108]
LncRNA-ID	RNA-seq	Machine-learning model	https://github.com/zhangy72/LncRNA-ID.git	[109]
lncRNA-MFDL	RNA-seq	Deep-learning classification algorithms	http://compgenomics.utsa.edu/lncRNA_MDFL/	[110]
uPEPperoni	RNA-seq	Codon substitution	http://upep-scmb.biosci.uq.edu.au	[111]
DeepCPP	RNA-seq	Machine-learning algorithms	https://github.com/yuuuuzhang/DeepCPP.git	[112]
RNAsamba	RNA-seq	Similarity to known proteins	https://rnasamba.lge.ibi.unicamp.br/	[113]
MipepID	Whole genome	Machine-learning algorithms	https://github.com/MindAI/MiPepid.git	[114]

**Table 4:** Association between SEP expression and diverse biological function.

Species	Symbol	Micro-Peptide	Length (aa)	Function	Category	Ref.
Human	LINC00948	MLN	46	Inhibits SERCA and regulates Ca^2+^ transport	Micro-peptide related muscle	[15]
Human	110017F19Rik /SMIM6	ELN	56			[146]
Human	1810037I17Rik	ALN	65			[146]
Human	LOC100507537	DWORF	34	Activates SERCA and regulates Ca^2+^ transport		[131]
Mouse	LOC101929726	Minion	84	Promotes myoblast fusion and muscle development		[147]
Mouse	LOC101929726	Myomixer	84			[16,148]
Fruit Fly	Scl	SclB	<30	Regulates Ca^2+^ transport		[149]
Chicken	Six ORF2	SIX1	74	Promotes cell proliferation and is involved in muscle growth		[142]
Mouse/ Zerbrefih	MyolncR4/ 1500011K16RIK	LEMP	56	Promotes muscle formation and regeneration in mice		[150]
Human	SPAR	LINC00961	75	Promotes muscle development		[17]
Human	miR-155HG	miPEP155 (P155)	17	Modulates antigen transport and presentation of antigen presenting cells	Micro-peptide related inflammation and immunity	[21]
Mouse	lncRNA 1810058I24Rik	Mm47	47	Controls innate immunity		[20]
Mouse	lncRNA Aw112010	Aw112010		Drives IL-12p40 production and mediates innate immune response		[22]
Human		IMP	_	Regulates inflammatory gene expression		[151]
Human /Mouse	LINC00116/ 1500011K16Rik	MOXI /Mitoregulin	56	Enhances mitochondrial β-oxidation	Micro-peptide related metabolism	[19]
Mouse	LINC00116	Mtln	56	Supports mitochondrial super-complexes and respiratory efficiency		[18]
Human	LINC-PINT	PINT87aa	87	Tumor suppressor in glioblastoma	Micro-peptides related tumors	[152]
Human	LINC00278	YY1BM	21	Promotes apoptosis and downregulates the survival rate of ESCC cells		[153]
Human	circFNDC3B	cirFND3B-218aa	218	Inhibits the expression of oncogene Snail and promotes CRC		[154]
Human	circPPP1R12A	PPP1R12A-C	73	Activates Hippo-YAP signaling pathway and inhibits CRC		[155]
Human	Meloe	MELOE-1	46	Involved in T cell immune surveillance; optimal T cell target for melanoma immunotherapy		[156]
Human		MELOE-2	39			[157]
Human	LINC00665	CIP2A-BP	52	Inhibits tumor invasion and metastasis		[158]
Human	LINC00998	SMIM30	59	Promotes cell proliferation and migration		[139]
Human	NCBP2-AS2	KRASIM	99	Inhibits carcinogenic signaling in hepatocellular carcinoma cells		[159]
Human	UBAP1-AST6	BAP1-AST6 (aa)	-	Promotes cell proliferation		[160]
Human	HOXB-AS3	HOXB-AS3	53	Inhibits cell proliferation, invasion, and metastasis		[161]
Human	CTD-2256P15.2	PACMP	44	Regulates cancer progression and drug resistance by modulating DNA damage response		[26]
Human	Rps41	RPS4XL	_	Inhibition of hypoxia-induced proliferation of pulmonary artery smooth muscle cells	Other diseases	[162]

## Data Availability

Publicly available datasets were analyzed in this study. This data can be found here: http://bigdata.ibp.ac.cn/SmProt/index.html and http://www.sorfs.org.
